# Sonographic imaging of the stellate ganglion in healthy adults: An observational study

**DOI:** 10.1097/MD.0000000000038646

**Published:** 2024-06-21

**Authors:** Mohamed A. Bedewi, Salvatore Marsico, Steven B. Soliman, Yomna S. Habib, Mamdouh Ali Kotb, Daifallah Mohammed Almalki, Ali Abdullah AlAseeri, Bader A. Alhariqi, Mohammed Saad Alqahtani, Anas Mohammad Albarrak, Ahmed Y. Alamir

**Affiliations:** a Department of Internal Medicine, Prince Sattam Bin Abdulaziz University, College of Medicine, Al-Kharj, Kingdom of Saudi Arabia; b Department of Radiology, Hospital del Mar, Barcelona, Spain; c Division of Musculoskeletal Radiology, Department of Radiology, University of Michigan, Ann Arbor, MI; d Department of Radiology, National Cancer Institute, Cairo University, Cairo, Egypt; e Minia University, Faculty of Medicine, Neuropsychiatry Department, Minya, Egypt; f Department of Pediatric Radiology Medical Imaging Administration, King Fahad Medical city, Kingdom of Saudi Arabia; g Radiology Department Faculty of Medicine, Cairo University, Cairo, Egypt.

**Keywords:** cross-sectional area, reference values, stellate ganglion, ultrasound

## Abstract

The aim of this study is to estimate the normal cross-sectional area and diameter of the stellate ganglion (SG) by ultrasound (US) in healthy adults. The study sample included 80 stellate ganglia in 40 participants (15 males, 25 females), mean age 38 years, mean height 162.5 cm, mean weight 67.8 kg, mean body mass index 25.4 kg/m^2^. Two radiologists separately obtained US images of the bilateral SG. Each participant was scanned 3 times bilaterally to assess for intra-observer reliability. The mean diameter of the SG was 1 mm (range: 0.1–2). The mean CSA of the bilateral SG was 1.3 mm^2^ (range: 0.6–3.9). The SG diameter positively correlated with age. Our study demonstrates the ability of US to image the SG and estimate its normal diameter and CSA. Knowledge of how to identify and measure the SG during ultrasound-guided procedures would be expected to decrease the risk of associated complications and help establish normal reference values.

## 1. Introduction

Nerve blocks of the peripheral nerves are increasingly used as an adjunct modality in a variety of diseases. Stellate ganglion (SG) blocks are one type of peripheral nerve blocks which are utilized in the treatment of conditions which are refractory to medical treatment. These conditions are mostly related to sympathetic mediated painful conditions and vascular insufficiency in the head, neck, and the upper limbs.^[1]^ The SG (also known as the cervicothoracic ganglion) is formed by the fusion of the first thoracic and inferior cervical ganglia. It lies anterior to the neck of the first rib and extends to the inferior surface of the transverse process of the seventh cervical vertebra. Sympathetic fibers supplying the head and neck organs, upper limb, thoracic viscera, and the heart connect to the SG.^[2–4]^ This explains the effective use of a SG block for the management of a myriad of pathologies associated with increased sympathetic activity of the peripheral nervous system including painful and nonpainful conditions of the head and neck and upper extremity.^[5]^ This procedure is commonly performed by regional anesthesiologists and pain medicine/management physicians.^[4]^ Several imaging modalities are used to localize the SG, including ultrasound (US), computed tomography, fluoroscopy, magnetic resonance imaging, and nuclear medicine. However, several factors limit the use of the majority of these modalities, including radiation exposure, high-cost relative to magnetic resonance imaging, and long acquisition times. The use of US is well known for pain management procedures with excellent visualization of the surrounding neurovascular structures and soft tissues^.[6]^ Furthermore, the added advantage of US dynamic imaging, in addition to a lower cost, higher resolution, lack of radiation, and portability are all benefits supporting the use of US for identifying the SG.^[5]^ Multiple complications are frequently reported following a SG block. These include hematomas, esophagitis, mediastinitis, and intravascular injection of local anesthetic into the vertebral artery.^[6]^ The close proximity of the SG to vital structures such as the carotid sheath and jugular vein, together with presence of other nearby important neurovascular structures of the neck renders this a challenging procedure. Knowledge of the anatomy as well as appropriate orientation of the US transducer in localizing the SG during blockage would be expected to decrease the complications resulting from injury of the surrounding vital structures.^[6,7]^ Inspite of the effectiveness of SG blockade procedure, failure to improve and persistence of symptoms is still reported by some patients. Safety and efficacy by identification of the ultrasound anatomy could be improved by identification of the SG and familiarity of its expected diameter and cross-sectional area (CSA).^[8,9]^ The aim of this study is to estimate the normal CSA and diameter of the SG by US in healthy adults.

## 2. Methods

### 2.1. Participants

After institutional review board approval (SCBR-043-2022), participants of the study were recruited between October 2022 and October 2023, and informed written consent was obtained. Volunteers were recruited from the employees of Prince Sattam Bin Abdulaziz University hospital. Inclusion criteria included male or female, greater than or equal to 20 years of age. Patients with prior neck surgery or prior neck radiation were excluded. For each participant, data including age, sex, body mass index (BMI), weight, and height were recorded.

### 2.2. Technique

**Figure 1.**
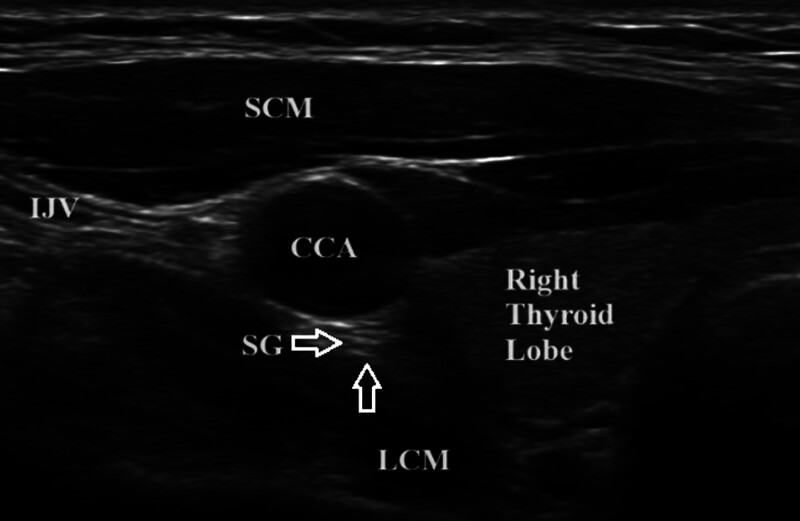
Short-axis view of the stellate ganglion, GAN = great auricular nerve, SCM = sternocleidomastoid muscle.

All US studies were performed utilizing an L12-5 MHz linear transducer (Epic 7 version1.5, Ultrasound system: Philips, Bothell, WA). All subjects were scanned separately by 2 radiologists; 1 (M.B) with 10 years’ experience in neuromuscular US, and the second (Y.S) with 3 years’ experience in neuromuscular US. Each participant was scanned 3 times with complete transducer removal from the skin following each measurement to assess for intra-observer reliability. In order to image the SG, all subjects were placed in a supine position with the neck in extension and the US transducer positioned in the transverse orientation to visualize the common carotid artery (CCA) and longus colli muscle (LCM) in the short-axis. The SG was imaged bilaterally in the short-axis and measurements were taken 3 separate times. The SG was identified as an oval hypoechoic structure between the CCA and the LCM, at the level of C7 and the first rib. The CSA of the SG was measured in mm^2^ using the tracer method by measuring inside the hyperechoic epineurium. The diameter was also measured in mm (Fig. 1).

### 2.3. Statistical analysis

Statistical analysis was performed using Statistical Package for the Social Sciences (SPSS) version 21 software (SPSS Inc, Chicago, IL). All data were presented as mean ± standard deviation (SD) and range. The mean CSAs were compared between both sides using Wilcoxon signed rank test. The correlations between the CSA of the imaged stellate ganglia, age, weight, height, and BMI were evaluated using Pearson correlation coefficient^®^. Paired sample *t* test was used to compare the CSA of the right and left SG. A *P* value of <.05 was considered significant.

## 3. Results

The study sample included 80 stellate ganglia in 40 participants (15 males (37.5%), 25 females), mean age 38.6 ± 12.7 years (range: 24–80), mean height 162.5 cm ± 10.9 (range: 145–195), mean weight 67.8 kg ± 18.1 (range: 45–135), mean BMI 25.4 kg/m^2^ ± 4.3 (range: 18.7–38). The intra-observer reliability calculations resulted in an overall intra-class correlation coefficient of 0.78. The mean diameter of the right SG was 1 mm ± 0.3 SD (range: 0.6–2). The mean CSA of the right SG was 1.2 mm^2^ ± 0.4 SD (range: 0.6–2.3). The mean diameter of the left SG was 0.9 mm ± 0.3 SD (range: 0.1–1.6). The mean CSA of the left SG was 1.4 mm^2^ ± 0.7 SD (range: 0.7–4).

There was no statistically significant difference when comparing the bilateral SG diameters (*P* = .193). There was also no statistically significant difference when comparing the bilateral SG CSAs (*P* = .312).

The mean diameter of the bilateral SG was 1 mm ± 0.3 SD (range: 0.1–2). The mean CSA of the bilateral SG was 1.3 mm^2^ ± 0.6 SD (range: 0.6–3.9). The diameter of the SG positively correlated with age (*P* < .001). Other demographic factors also demonstrated no statistically significant correlation (Tables 1 and 2).

**Table 1:** Demographic characteristics of study participants (mean ± standard deviation).

Descriptive statistics					
	N	Minimum	Maximum	Mean	Std. deviation
Age	40	24	80	38.6	12.7
Weight in kg	40	45.0	135.0	67.8	18.1
Height in cm	40	145.0	195.0	162.5	10.9
BMI	40	18.70	38.00	25.4	4.3

BMI = body mass index, SG = stellate ganglion.

**Table 2:** CSA, diameter of the SG, (mean ± standard deviation).

	N	Minimum	Maximum	Mean	Std. Deviation
Diameter if the right SG in mm	40	0.6	2	1	0.3
CSA of the right SG	40	0.6	2.3	1.2	0.4
Diameter if the left SG in mm	40	0.1	1.6	0.9	0.3
CSA of the left SG	40	0.7	4	1.4	0.7
Valid N (list wise)	40				

CSA = cross-sectional area, BMI = body mass index, SG = stellate ganglion.

## 4. Discussion

In this study we investigated the use of US of the SG to measure the diameter and CSA. Our results in general showed comparatively lower SG CSAs and diameters than those reported in the prior literature. However, we found only one study in the literature reporting the CSA of the SG by US. In that study, Li et al^[9]^ reported a CSA of 14.08 ± 4.42 mm^2^ (range: 5–27 mm^2^), and a diameter of 5.42 ± 0.95 mm (range: 3.6–7.7 mm). Multiple cadaveric studies have been performed measuring the diameter of the SG. In general, they reported higher values compared to our results as follows: Kiray et al (2.2 ± 0.7); Saylam et al (8.1 ± 2.8, range: 3.5–15.6 mm); Zhang et al (6.3 mm); and Marcer et al (8.2 mm).^[10–16]^ Hogan et al^[16]^ evaluated the SG by MRI imaging in 9 volunteers and although they were able to demonstrate the SG in all subjects, they reported variable shapes with a maximum cephalocaudal dimension of 1 cm. We found no difference between the CSA on either side which coincided with the findings of Li et al. We also found no difference in diameter between both sides, which is contrary to the findings of Li et al^[9]^ who reported that the diameter of the right SG was significantly higher than the left. Our study was the first to show that the diameter of the SG positively correlated with age. The difference in size in our study may be attributed to difference in study cohorts as well the difference in defining the margins of the SG. US of the SG and reporting sizes can be challenging due to multiple factors. First, knowing the level it is imaged and its variability in shape, including stare-shaped and oval/globular-shaped. Second, poor identification of the lower pole^.[12,17,18]^ Third, whether measurements are taken in the short-axis only or in both the short- and long-axes. Fourth, studies using different cohorts. Fifth, the type of US system used to image the SG. Sixth, inter-observer variability and differences in experience levels with neuromuscular US. Our study has several limitations which should be considered when interpreting the results. First, there were a limited number of subjects enrolled, however, this was similar or more than prior studies. The small sample size will definitely limit the implications of our results. Second, only using the CCA and LCM as the main markers for identification of the SG with optional visualization of C7. Further studies with increased sample size and added parameters would give more strength to the results. In conclusion, our study demonstrates the ability of US to image the SG and define its diameter and CSA. Knowledge of how to identify and measure the SG during ultrasound-guided procedures would be expected to decrease the risk of associated complications and help establish normal reference values.

## Acknowledgments

The authors thank the Deanship of Scientific Research at Prince Sattam bin Abdulaziz University.

## Author contributions

**Conceptualization:** Mohamed Abdelmohsen Bedewi, Bader A. Alhariqi.

**Investigation:** Mohamed Abdelmohsen Bedewi, Salvatore Marsico, Yomna S. Habib.

**Methodology:** Mohamed Abdelmohsen Bedewi, Salvatore Marsico, Yomna S. Habib, Mamdouh Ali Kotb.

**Project administration:** Mohamed Abdelmohsen Bedewi, Anas Mohammad Albarrak.

**Supervision:** Mohamed Abdelmohsen Bedewi, Daifallah Mohamed Almalki, Ali Abdullah AlAseeri, Bader A. Alhariqi, Mohammed Saad Alqahtani, Anas Mohammad Albarrak.

**Validation:** Mohamed Abdelmohsen Bedewi, Daifallah Mohamed Almalki.

**Visualization:** Mohamed Abdelmohsen Bedewi, Ahmed Y. Alamir.

**Writing – original draft:** Mohamed Abdelmohsen Bedewi, Steven B. Soliman.

**Writing** – **review & editing:** Mohamed Abdelmohsen Bedewi, Steven B. Soliman.

**Formal analysis:** Mamdouh Ali Kotb.

**Resources:** Mamdouh Ali Kotb, Ali Abdullah AlAseeri, Bader A. Alhariqi, Mohammed Saad Alqahtani.
